# Application of the deep learning algorithm in nutrition research – using serum pyridoxal 5′-phosphate as an example

**DOI:** 10.1186/s12937-022-00793-x

**Published:** 2022-06-10

**Authors:** Chaoran Ma, Qipin Chen, Diane C. Mitchell, Muzi Na, Katherine L. Tucker, Xiang Gao

**Affiliations:** 1 Channing Division of Network Medicine, Brigham and Women’s Hospital and Harvard Medical School, Boston, MA USA; 2 Department of Mathematics, The Pennsylvania State University, University Park, State College, PA USA; 3 Department of Nutritional Sciences, The Pennsylvania State University, University Park, State College, PA USA; 4 Department of Biomedical & Nutritional Sciences, The University of Massachusetts at Lowell, Lowell, MA USA; 5 Department of Nutrition and Food Hygiene, School of Public Health, Fudan University, 130 Dongan Rd, Shanghai, China

**Keywords:** Pyridoxal 5′-phosphate, Vitamin B6, Dietary pattern, Deep learning, NHANES, Multivariable linear regression

## Abstract

**Background:**

Multivariable linear regression (MLR) models were previously used to predict serum pyridoxal 5′-phosphate (PLP) concentration, the active coenzyme form of vitamin B6, but with low predictability. We developed a deep learning algorithm (DLA) to predict serum PLP based on dietary intake, dietary supplements, and other potential predictors.

**Methods:**

This cross-sectional analysis included 3778 participants aged ≥20 years in the National Health and Nutrition Examination Survey (NHANES) 2007-2010, with completed information on studied variables. Dietary intake and supplement use were assessed with two 24-hour dietary recalls. We included potential predictors for serum PLP concentration in the models, including dietary intake and supplement use, sociodemographic variables (age, sex, race-ethnicity, income, and education), lifestyle variables (smoking status and physical activity level), body mass index, medication use, blood pressure, blood lipids, glucose, and C-reactive protein. We used a 4-hidden-layer deep neural network to predict PLP concentration, with 3401 (90%) participants for training and 377 (10%) participants for test using random sampling. We obtained outputs after sending the features of the training set and conducting forward propagation. We then constructed a loss function based on the distances between outputs and labels and optimized it to find good parameters to fit the training set. We also developed a prediction model using MLR.

**Results:**

After training for 10^5^ steps with the Adam optimization method, the highest *R*^*2*^ was 0.47 for the DLA and 0.18 for the MLR model in the test dataset. Similar results were observed in the sensitivity analyses after we excluded supplement-users or included only variables identified by stepwise regression models.

**Conclusions:**

DLA achieved superior performance in predicting serum PLP concentration, relative to the traditional MLR model, using a nationally representative sample. As preliminary data analyses, the current study shed light on the use of DLA to understand a modifiable lifestyle factor.

**Supplementary Information:**

The online version contains supplementary material available at 10.1186/s12937-022-00793-x.

## Background

Vitamin B6 plays vital roles in numerous metabolic processes in the human body, including in the hematologic, cardiovascular, and neurologic systems [1]. Pyridoxal 5′-phosphate (PLP) is an active coenzyme form of vitamin B6, functioning as an essential cofactor and regulator for various enzyme-catalyzed reactions [2]. However, although vitamin B6 status has been shown to be closely related to dietary vitamin B6 intake in a laboratory setting [3], in previous epidemiological studies, due partially to the nature of variation in diet and measurement error of dietary assessment [4], dietary intakes of vitamin B6 and foods rich in vitamin B6 explained only a small portion of the variance in serum PLP [5–7]. Knowing that dietary intake is complex, it has been generally acknowledged that there is a need to develop and refine methods of assessing dietary intake, focusing on the overall dietary pattern [8]. Examining the effects of overall diet takes into account nutrient interactions and allows for capturing diet-biomarker relations without particular knowledge of the specific nutrient or food component involved [9].

Traditionally, multivariable linear regression (MLR) models are used for prediction in the health field, assuming linear relationships between all predictors and the response variable. However, the human body is such a complex organism that the linear model may not provide the best fit. In contrast, machine learning technology, one of the major approaches for artificial intelligence research, uses stunningly complicated networks of artificial neurons, designed expressly to create accurate models directly from raw data, being able to learn the task with little human instruction or prior assumptions. By optimizing loss functions, models find their weights or parameters automatically. According to the depths of the models, machine learning can be divided into two categories: “shallow” learning and deep learning. Due to the restriction of structures of “shallow” learning, deep learning has demonstrated better performances on different kinds of tasks, such as computer vision and language processing [10, 11]. Recently, researchers have applied the deep learning technology to diabetic retinopathy screening [12], detection of lymph node metastases from breast cancer [13], identification of tuberculosis patterns [14], classification of skin cancer [15], and food image recognition in nutrition [16–18]. However, those algorithms, focused on disease diagnosis and screening, were generally image-based [12–15]. To our knowledge, the feasibility of using deep learning technology for health outcomes in relation to modifiable lifestyle factors, such as nutritional factors, has not yet been investigated.

In this context, we developed a deep learning algorithm (DLA) to predict serum PLP concentration based on 1) dietary intake and dietary supplement use, to create a dietary pattern; and 2) further including sociodemographic information (age, sex, race/ethnicity, education level, and the ratio of family income to poverty), lifestyle factors (smoking status and physical activity), and other non-dietary variables (anti-hypertension medication use, cholesterol-lowering medication use, insulin treatment, anti-diabetes medication use, systolic blood pressure, diastolic blood pressure, glucose, glycosylated hemoglobin, body mass index, high-density lipoprotein cholesterol, low-density lipoprotein cholesterol, total cholesterol, and C-reactive protein) to maximize the prediction in a sample of 3778 U.S. adults from the National Health and Nutrition Examination Survey (NHANES). We also developed an MLR model, using the same variables as used with DLA, and compared the *R*^*2*^ values between results using DLA and MLR for serum PLP prediction.

## Methods

### Study participants

The NHANES is a cross-sectional nationwide survey to assess health and nutritional status of the noninstitutionalized U.S. population [19]. The current study was based on the NHANES 2007 to 2010 samples, because different information was collected in each survey cycle, and these were the most recent cycles that had released the variables of interest. Analyses for this study were limited to adults aged 20 years and older, which the NHANES had set as the age restriction for participants to receive adult-specific questionnaires. After excluding participants with missing information on variables of interest (i.e., serum PLP concentration and potential predictors), *n* = 3778 participants were included in the current study (Supplemental Fig. 1). NHANES was approved by the Institutional Review Board of the National Center for Health Statistics. Informed consent was obtained from all participants.

### Assessment of serum PLP (outcomes)

Serum vitamin B6, in the form of PLP, was measured by investigators at NHANES using reversed-phase high-performance liquid chromatographic (HPLC) with fluorometric detection at 325 nm excitation and 425 nm emission. Because chlorite post-column derivatization could oxidize PLP to a more fluorescent carboxylic acid form, post-column introduction of a sodium chlorite derivatization reagent was incorporated into the HPLC system to improve the PLP signal [20]. Quantification was based on analyte peak area interpolated against a five-point calibration curve obtained from aqueous standards. The mean coefficient of variation for the assay was 4.9% and the detection limit of the assay was 0.3 nmol/L [21].

### Assessment of potential predictors

Information on dietary intake was obtained using two 24-h dietary recall interviews. The first 24-h recall interview was conducted in-person in the NHANES Mobile Examination Center (MEC) at the same timepoint with examination components and biospecimen collection, and the second day was collected by telephone 3 to 10 days later. Two well-trained dietary interviewers administered the dietary interview at each MEC comprising three sections: (a) dietary recall, (b) nutritional supplement and antacid use, and (c) post-recall [22]. Average dietary intake, based on the 2 days, were used in the current analysis. The U.S. National Center for Health Statistics was responsible for the sample design and data collection and U.S. Department of Agriculture (USDA) Food Surveys Research Group was responsible for the dietary data collection methodology, maintenance of the databases used to code and process the data, and data review and processing [23]. The foods and beverages in the dietary interview components were converted to the 37 USDA food groups (Supplemental Table 1), based on the Food Patterns Equivalents Database (FPED). The FPED served as a unique research tool to evaluate foods and beverage intakes of Americans with respect to the 2015-2020 Dietary Guidelines for Americans [24].

Information on dietary supplement use was collected after the 24-h dietary recall for foods and beverages, using a similar protocol. Information was obtained on all vitamins, minerals, herbals, and other dietary supplements as well as non-prescription antacids that were consumed during a 24-h time period (midnight to midnight), including the name and the amount of supplement taken. Daily vitamin B6 supplement intake was calculated using the NHANES Dietary Supplement Database [25].

Demographic variables (age, sex, race/ethnicity, education level, and the ratio of family income to poverty), lifestyle factors (smoking status and physical activity), and information on medication use were derived from questionnaires in the home by trained interviewers, using the Computer-Assisted Personal Interviewing system. Education level was the highest grade completed by the participant, and was described as < 12 years (middle and elementary school), 12 years (high school) and > 12 years (college and graduate School). The income-to-poverty ratio reflected the ratio of an individual’s household income to the federal poverty level, adjusted for household size and composition [26]. Smoking status was categorized as never, former, or current smoking. Physical activity was categorized as below (< 150 minutes per week of moderate-intensity), meeting (150-299 minutes per week of moderate-intensity), or exceeding (≥300 min per week of moderate-intensity) the federal physical activity guideline recommendations [27]. Medication use included antihypertensive, antiglycemic, cholesterol-lowering agents and use of insulin (Yes/No for each). Systolic and diastolic blood pressures were measured three times from the seated position. If a blood pressure measurement was interrupted or incomplete, a fourth attempt was made [28]. The average of all available readings was used for analysis. Body mass index (BMI) was calculated as body weight (kg) divided by the square of height (m^2^). Blood triglycerides, high-density lipoprotein cholesterol (HDL-C), low-density lipoprotein cholesterol (LDL-C), and fasting plasma glucose were measured using a Roche Modular P chemistry analyzer. Glycosylated hemoglobin was measured on an A1c G7 HPLC Glycohemoglobin Analyzer (Tosoh Medics, Inc., 347 Oyster Pt. Blvd., Suite 201, So. San Francisco, Ca 94,080.). C-reactive protein assays were performed on a Behring Nephelometer [29].

### Statistical analyses

Statistical analyses and all the computations for the current study were conducted with SAS 9.4 (SAS Institute Inc., Cary, NC) and Python 3.5 (Python Software Foundation, Delaware City, DE). The deep neural network structure was constructed with PyTorch (Adam Paszke, Sam Gross, Soumith Chintala, Gregory Chanan) [30]. PyTorch is an open source machine learning library for Python, used for applications such as computer vision and natural language processing. PyTorch has Graphical Processing Unit (GPU) support on tensor computation and an automatic gradients computing system [31].

Simple random sampling was used to divide the labeled dataset into training and test datasets with PROC SURVEYSELECT in SAS. The training set is a dataset of examples used for learning. By constructing a loss function based on the training set and finding a local (even global) minimum of the loss function, we can obtain good parameters or weights in the network to fit the training set. We assume that the test set followed the same probability distribution as the training set; if our model fits the training set, it also should fit the test set well. In general, there is no clear criterion for the ratio of training and test datasets. Samples are usually split, based on the data quality and sample size, with different ratios; the ratio of 90%:10% has been commonly used in previous studies [32, 33]. We held out 10% of the labeled dataset as the test dataset, which was used to determine the final model performance, and thus excluded from model development or tuning. In order to obtain a stable prediction model, we trained and selected a model using the remaining 90% as training data. Once the final model had been selected, we tested the performance on the 10% test sample, using *R*^*2*^ as the proportion of the variance in the dependent variable that was predictable from the independent variables. *R*^*2*^ is a function of the total sum of squares (SST) and the sum of squared errors (SSE) (${R}^2=1-\frac{SSE}{SST}$). For both the DLA and MLR models, we developed two models: first, including food groups and vitamin B6 supplement intake and, second, including food groups, vitamin B6 supplement intake, and other aforementioned potential non-dietary predictors.

#### DLA predication model

**Fig. 1:**
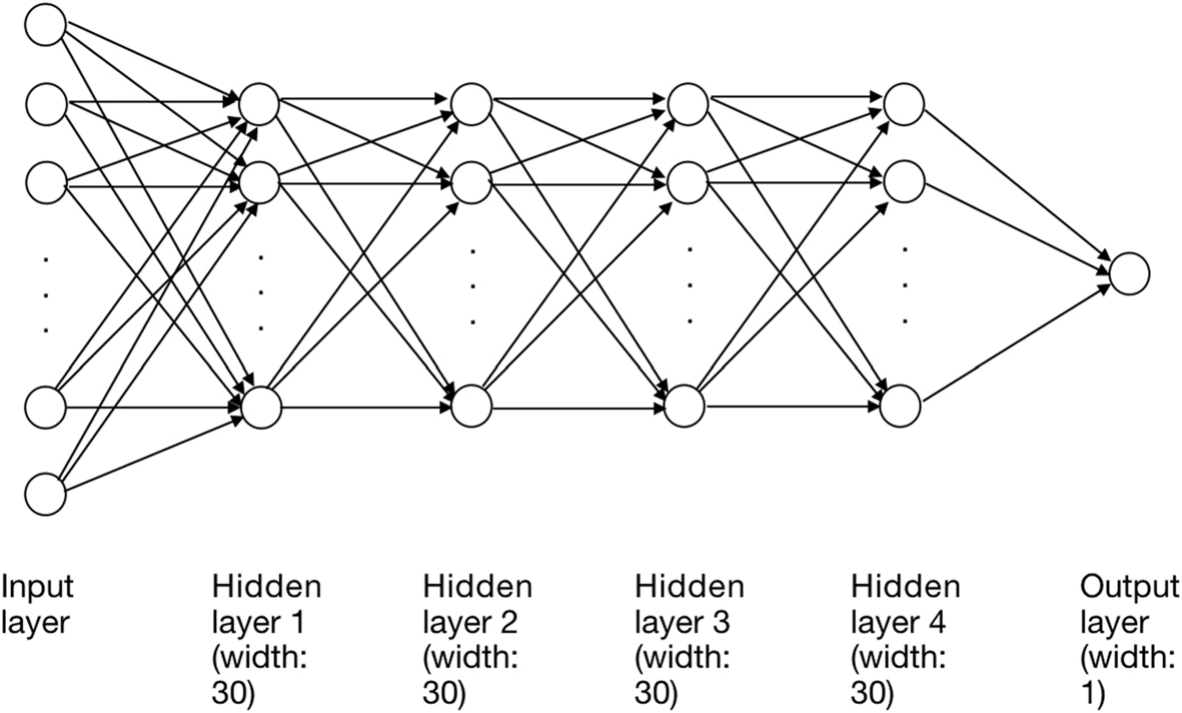
The structure of neural network

Deep neural networks are a class of models within the machine learning area which identify a nonlinear relationship between the input, x, and the output y [27]. Normally there are three types of layers in neural networks, the input layer, the output layer and the hidden layer (see Fig. 1 for an example). With an appropriate number of hidden layers, with certain nodes for each hidden layer, the neural network can be used to approximate the nonlinear function, *y* ≈ *f*(*x*). In our DLA model, we used a 4-hidden-layer fully connected neural network with the width of 30 nodes for each layer. Each neuron was connected by all the neurons in the previous layer (Fig. 1). In particular, the mathematical expression of the DLA model is following:

$$f\left(x;P\right)={W}_5\sigma \left({W}_4\sigma \left({W}_3\sigma \left({W}_2\sigma \left({W}_1x+{\theta}_1\right)+{\theta}_2\right)+{\theta}_3\right)+{\theta}_4\right)+{\theta}_5,$$

where x is the input data, P is the parameter set, namely, *P* = {*W*_*i*_, *θ*_*i*_}, *i* = 1, ⋯5, and *σ*(*x*) is the rectified linear unit (ReLU) activation function [34] which has the form of the following form:

$$\upsigma \left(\mathrm{x}\right)=\max \left(x,0\right)$$

Theoretically speaking, this neural network setup can approximate any dependencies between the input and the output, when the number of layers and nodes is large enough [35]. In particular, when the data have nonlinear dependencies, neural networks are able to perform better than regression, which is designed to reconstruct only linear dependencies and to ignore the nonlinearities. Moreover, regression models can be recovered by a simple neural network which only involves the input and output layers but no hidden layers.

To find the optimal parameter set, we needed to solve an optimization problem to minimize the distance between the empirical data and the model prediction, namely,

$$\underset{P}{\min }L(P)\triangleq \frac{1}{n}\sum_{i=1}^n{\left|f\left({x}_i,P\right)-{y}_i\right|}^2,$$

which is the loss function in machine learning. Here {(*x*_*i*_, *y*_*i*_)| *i* = 1, ⋯*n*} includes the training data and labels, and n is the number of participants in the training set. To solve this optimization problem, we employed the Adam algorithm [36], an algorithm for first-order gradient-based optimization of stochastic objective functions, based on adaptive estimates of lower-order moments, as our optimization method. We chose 0.001 as the learning rate to prevent overshooting which means wandering around the lowest point, because the learning rate was too high for the model when applying a gradient-based optimization algorithm. To prevent overfitting, we used batch normalization [25] and dropout [37], with a probability of 0.5, as regularization—a method to prevent overfitting by adding the norm of weight parameters to the loss function.

#### MLR prediction model

Because we were only interested in the description of samples without making any inferential conclusion in this study, we developed two MLR prediction models using the training data, including dietary and non-dietary predictors, as detailed above. To compare with the DLA models, we did not exclude any potential predictors in the MLR model based on their significant levels.

#### Sensitivity analyses

To test the robustness of our results, we conducted two sensitivity analyses. Because vitamin B6 supplement intake was strongly correlated with serum PLP concentration, we conducted subgroup analysis, stratified by vitamin B6 supplement use status (yes/no). We then included only variables identified by a stepwise regression model of the MLR (*p* = 0.5 for entry, and *p* = 0.1 for removal) in the DLA prediction model. Stepwise regression is a modification of the forward selection and backward elimination technique. As in the forward selection technique, variables are added one at a time to the model, as long as the F statistic *p*-value is below the specified α. After a variable is added, however, the stepwise technique evaluates all of the variables already included in the model and removes any variable that has an insignificant F statistic p-value exceeding the specified α. Only after this check is made and the identified variables have been removed can another variable be added to the model. The stepwise process ends when none of the variables excluded from the model has an F statistic significant at the specified α and every variable included in the model is significant at the specified α.

## Results

The mean age of the 3778 participants was 50.7 years, and 53.0% were women. Mean ± standard error (SE) of serum PLP concentration was 65.6 ± 1.15 nmol/L, and 31.4% of participants used supplements that contained vitamin B6. Characteristics were similar between the training and test groups (*P* > 0.05 for all) (Table 1).

**Table 1:** Descriptive characteristics of participants in training and test datasets in U.S. adults^a^

	Training	Test	*P* value
*n*	3401	377	
Age, y	50.7	50.9	0.80
Women, %	53.5	49.1	0.10
Education, %			0.48
Less than high school (< 12 years)	27.3	24.9	
Completed high school (12 years)	23.3	24.7	
More than high school (> 12 years)	49.4	50.4	
Ethnicity, %			0.34
Hispanics	23.1	27.0	
Non-Hispanic White	55.2	52.2	
Non-Hispanic Black	18.6	16.9	
Other races	3.2	3.9	
Ratio of family income to poverty	2.57	2.48	0.35
Adherence to physical activity guideline recommendations, %			0.33
Below (< 150 minutes a week of moderate-intensity)	11.3	13.0	
Meeting (150-299 minutes a week of moderate-intensity)	40.6	40.6	
Exceeding (≥300 minutes a week of moderate-intensity)	48.1	46.4	
Smoking status, %			0.37
Never smoking	54.3	54.9	
Former smoking	25.9	28.7	
Current smoking	19.9	16.5	
Anti-Hypertension medication use, %	32.2	34.5	0.43
Cholesterol-lowering medication use, %	19.2	19.4	0.91
Insulin treatment, %	2.7	4.2	0.11
Anti-Diabetes medication use, %	9.9	11.4	0.41
Systolic blood pressure, mm/Hg	122	124	0.06
Diastolic blood pressure, mm/Hg	68	68	0.89
Glucose^b^, mg/dL	108	108	0.74
Glycohemoglobin, %	5.72	5.69	0.67
Body mass index, kg/m^2^	29.0	28.7	0.46
High density lipoprotein cholesterol^c^, mg/dL	53.7	54.2	0.49
Low density lipoprotein cholesterol^c^, mg/dL	115.8	115.4	0.84
Total cholesterol^c^, mg/dL	194.4	194.8	0.86
C-reactive protein^d^, mg/dL	0.41	0.41	0.90
Daily vitamin B6 supplement, mg/d	3.73	3.59	0.89
Serum pyridoxal 5′-phosphate, nmol/L	65.6	66.6	0.81
Total energy intake, kcal	2019	2005	0.71

^a^Values are mean (standard error) adjusted for age and sex, or percentages

^b^The fasting glucose value in mg/dL can be converted to mmol/L by multiplying by 0.05551

^c^The cholesterol value in mg/dL can be converted to mmol/L by multiplying by 0.02586

^d^The C-reactive protein value in mg/dL can be converted to mg/L by multiplying by 10

**Fig. 2:**
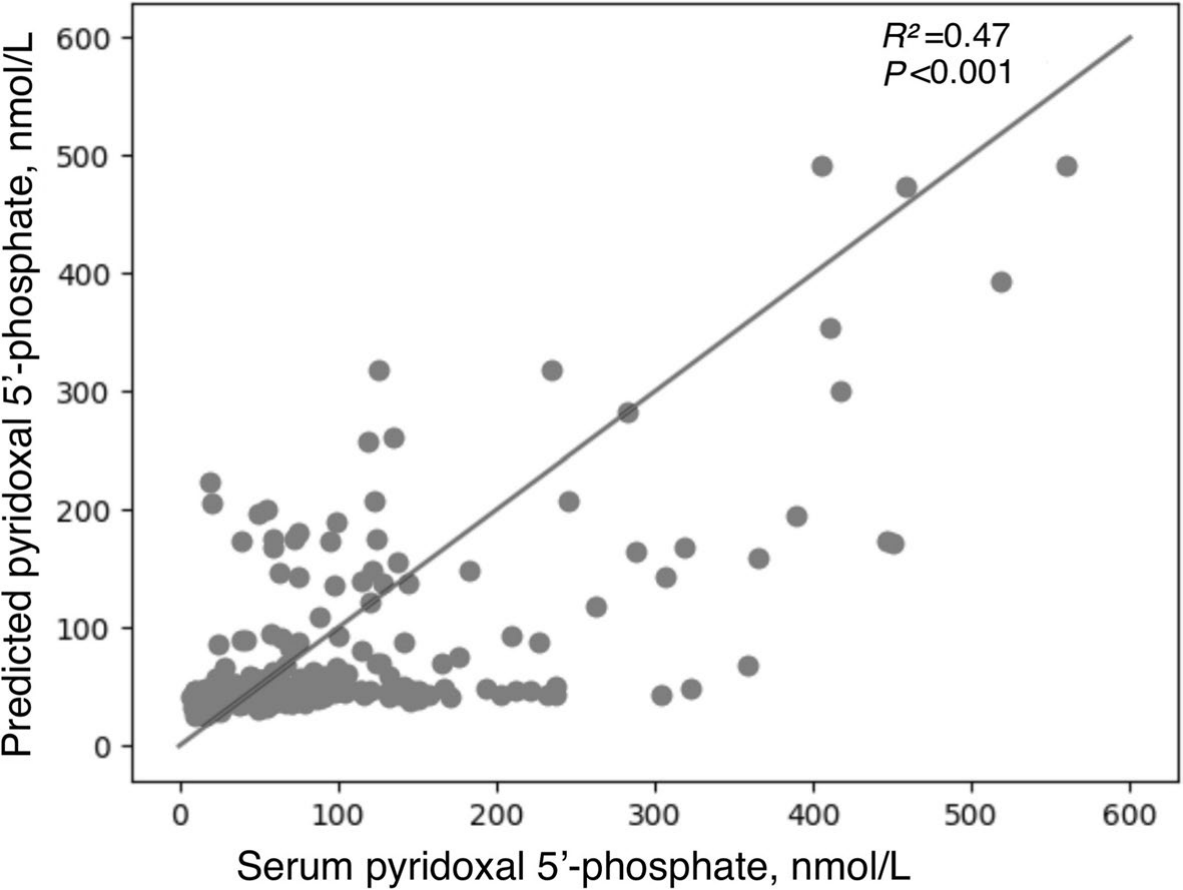
The relationship between serum pyridoxal 5′-phosphate concentration and predicted pyridoxal 5′-phosphate value based on deep learning algorithm (DLA)

After training for 10^5^ steps with the Adam optimization method, the highest *R*^*2*^ was 0.41 for the DLA and 0.15 for the MLR model in the test dataset using 37 food groups and vitamin B6 supplement use as predictors (Table 2). The *R*^*2*^ was improved slightly after further including other potential non-dietary predictors: the corresponding *R*^*2*^ was 0.47 for DLA (Fig. 2) and 0.18 for MLR in the test dataset (Table 2). Similar results remained in the subgroup analysis, stratified by supplement use status, and sensitivity analyses including variables identified by a stepwise regression model (Table 2).

**Table 2:** R squares for pyridoxal 5′-phosphate prediction models, based on deep learning algorithm versus multivariable linear regression

	37 Food groups and supplement variables included^a^				Further including non-dietary variables^b^			
	Deep learning algorithm		Multivariable linear regression		Deep learning algorithm		Multivariable linear regression	
	Training	Test	Training	Test	Training	Test	Training	Test
All participants	0.46	0.41	0.21	0.15	0.43	0.47	0.25	0.18
Excluding users of vitamin B6 supplements	0.36	0.33	0.08	0.08	0.49	0.33	0.15	0.16
Including only users of vitamin B6 supplements	0.59	0.53	0.20	0.17	0.66	0.51	0.25	0.21
Including only variables identified by the stepwise model^d,e^	0.45	0.41	0.21	0.15	0.52	0.38	0.25	0.18

^a^Variables include energy intake, vitamin B6 supplement, citrus/melons/and berries, other fruits, fruit juice, dark green vegetables, tomatoes, other red and orange vegetables, potatoes, other starchy vegetables, other vegetables, beans and peas (vegetables), whole grains, refined grains, meat, cured meat, organ meat, poultry, seafood high in n-3 fatty acids, seafood low in n-3 fatty acids, eggs, soy products, nuts and seeds, beans and peas (proteins), milk, yogurt, cheese, oils, solid fats, added sugars, and alcoholic drinks

^b^Variables in^1^ and also age, sex, education, ethnicity, ratio of family income to poverty, adherence to physical activity guideline recommendations, smoking status, anti-hypertension medication use, cholesterol-lowering medication use, insulin treatment, anti-Diabetes medication use, systolic blood pressure, diastolic blood pressure, glucose, glycosylated hemoglobin, body mass index, high-density lipoprotein cholesterol, low-density lipoprotein cholesterol, total cholesterol, and C-reactive protein

^c^Variables for 37-food groups, include added sugars, alcoholic drinks, cheese, milk, yogurt, fruit juice, other fruits, whole grains, oils, cured meat, legumes (proteins), nuts and seeds, poultry, seafood high in n-3 fatty acids, soy products, solid fats, legumes (vegetables), other vegetables, other red and orange vegetables, tomatoes, other starchy vegetables, and vitamin B6 supplement

^d^Variables for the dietary and non-dietary model include age, sex, ratio of family income to poverty, smoking status, systolic blood pressure, cholesterol-lowering medication use, glucose, body mass index, high-density lipoprotein cholesterol, C-reactive protein, alcoholic drinks, mile, yogurt, fruit juice, other fruits, refined grains, whole grains, oils, cured meat, legumes (proteins), nuts and seeds, poultry, seafood high in n-3 fatty acids, soy products, solid fats, legumes (vegetables), other vegetables, other red and orange vegetables, tomatoes, other starchy vegetables, and vitamin B6 supplement

## Discussion

Using data from a nationally representative sample of American adults, we developed a DLA to predict serum PLP concentration based on dietary intake, vitamin B6 supplement use, and other non-dietary factors. Compared with the traditional prediction model from MLR, DLA resulted in *R*^*2*^ with twice as high.

Although we know of no published study using dietary patterns to predict serum PLP, the association between dietary vitamin B6 intake and serum PLP has been investigated previously. For example, one cross-sectional study (*n* = 198, mean age, 72 years) showed that vitamin B6 supplementation, but not dietary vitamin B6, was significantly associated with serum PLP concentration [5]. Another cross-sectional study, including 1239 Puerto Rican adults, examined associations between consumption of 15 vitamin B6 rich foods, vitamin B6 supplements and serum PLP concentration. Only vitamin B6 supplements and ready-to-eat cereal were found to be significantly associated with PLP [7]. In the current study, we examined the overall impact of dietary patterns on serum PLP concentration, not only to reinforce the concept proposed in the latest American Dietary guidelines [38], but also to capture as much information as possible because vitamin B6 is present in many foods.

In the current study, the *R*^*2*^ of DLA for predicting PLP concentration was 0.47 in the test dataset, two times as high as that of MLR, which is traditionally used for prediction. Some mathematical proofs of the approximation property of DLA could explain the reason why the DLA demonstrated better performances than the MLR. For instance, when any continuous function can be approximated by DLA with only one hidden layer, then the ability of fitting sample points of training set is guaranteed theoretically. MLR can only fit the linear distribution, which may be not suitable when the distribution of sample points is more complicated. As the application of artificial intelligence has been grown in the health field, there has been surprise at the extraordinarily performance of the state-of-the-art technology. However, to our knowledge, these technologies have been mostly adopted to recognize images and make classifications. For example, the first study using DLA to detect referable diabetic retinopathy came out at the end of 2016 [12]. In this novel study, a deep convolutional neural network was trained using 128,175 retinal images, which were graded 3 to 7 times by a panel of 54 U.S. licensed professionals, resulting in a mean area under the receiver operating curve of 0.99, with high sensitivity and specificity (sensitivity ranged from 90.3 to 97.5%; specificity ranged from 93.4 to 98.5%) [12]. The results were straightforward because the images already contained necessary information required for the classification. The function computed disease severity from the intensities of the pixels in an image. In nutrition, deep convolutional neural networks have been used in the field of food image recognition to estimate food intake [16–18], serving as an alternative or complementary approach to traditional questionnaire-based dietary assessment. Meanwhile, research on predicting a variable affected by various known and even unknown factors using artificial intelligence has just begun. More recently, applications of deep learning using data drawn by a commercially available device (e.g., smartwatch, and smartphone) were conducted [39, 40]. Compared with image-based studies, those data-based studies showed more modest accuracy, with a sensitivity and specificity below 70% for detection of atrial fibrillation [39] and a correlation of 0.81 for quantifying Parkinson disease severity [40], because of variability and noise in the data. Unlike some nutrients, either with limited food sources or in specific fortified food products (e.g., vitamin B12 or vitamin D), vitamin B6 is found in a wide variety of foods [41]. The diverse food sources of vitamin B6 increases the difficulty in accurate estimation of dietary intake, thus increasing the difficulty in the prediction of serum status. In this context, we used serum PLP as an example to investigate the feasibility of the technology.

A main strength of this study is that it is the first study to have applied artificial intelligence in the field of nutrition assessment, shedding light on the importance of modifiable diet factors for prevention of diseases due to low vitamin B6 status. This is also the first study to examine the association between dietary patterns and serum PLP, reinforcing the concept of emphasizing overall eating pattern in the latest American dietary guidelines [38]. Additionally, the rich NHANES data set provided a valuable opportunity to perform analyses that include a comprehensive set of covariates, both to clarify findings and control bias.

On the other hand, the current study also has some limitations. First, approximately half of the variation in serum PLP was still not explained by the DLA model. For example, for low measured serum PLP (< 20 nmol/L), the predicted PL*P* values varied over a large range. However, even so, using DLA resulted in twice the *R*^*2*^ value compared with the traditional prediction model using MLR. Moreover, there are inherent disadvantages in using two 24-h dietary recalls as assessment of usual food intake for the participant [4]. To reduce the potential misclassification, dietary intakes in NHANES were assessed on two non-consecutive days by experienced and well-trained interviewers using a standardized protocol, capturing more information on the day-to-day variation than a single 24-hour dietary recall. In addition, because genetic information was not available for this study, it is unknown how the predictability would change after integration of PLP-related genetic factors. Moreover, although our sample size was 3778, it was still considered relatively small for conducting deep learning technology, which requires a large data set. In general, there is no defined principle for sample size selection. A sample size larger than the number of parameters is acceptable and the more parameters there are, the larger the sample size required. In the current study, the structure of our DLA was not overwhelmingly complicated, indicating that the sample size in our experiment was sufficient to generate acceptable results. Finally, there are limitations in generalizability to other settings and populations. The prediction model needs to be replicated in an independent external population. However, as a preliminary analysis, these results provide valuable and relevant data in support of a new application of artificial intelligence for a modifiable lifestyle factor.

## Conclusions

DLA achieved superior performance in predicting serum PLP concentration, compared to the traditional MLR model, supporting the feasibility of using artificial intelligence in nutrition research. Future studies using DLA with larger sample size, genetic information, and improved algorithms are warranted. Given that healthy lifestyles, including dietary patterns, can help people achieve and maintain good health and reduce the risk of chronic disease throughout all stages of the lifespan, the DLA approach may help to more accurately identify modifiable lifestyles variables at large scale, thereby clarifying opportunities for intervention to improve nutrition and public health.

## Supplementary Information

**Additional file 1: Supplemental Figure 1.** Participant Flow Chart. **Supplemental Table 1.** Food Patterns Equivalents Database Components.

## Data Availability

The datasets generated and/or analyzed during the current study are available at https://wwwn.cdc.gov/nchs/nhanes/default.aspx.

## Abbreviations

DLA: Deep learning algorithm
FPED: Food Patterns Equivalents Database
HPLC: High-performance liquid chromatographic
MLR: Multivariable linear regression
NHANES: National Health and Nutrition Examination Survey
PLP: Pyridoxal 5′-phosphate
USDA: United States Department of Agriculture
