# Supplementary material for: Application of the deep learning algorithm in nutrition research – using serum pyridoxal 5′-phosphate as an example
Source: Nutr J. 2022 Jun 10;21:38. doi: 10.1186/s12937-022-00793-x (PMC9185886; doi:10.1186/s12937-022-00793-x)
Supplement: Supplementary file 1 — Additional file 1: Supplemental Figure 1. Participant Flow Chart. Supplemental Table 1. Food Patterns Equivalents Database Components. [file 12937_2022_793_MOESM1_ESM.docx]

**Online-only Supplements**

**Tables: 1**

**Figure: 1**

Supplemental Figure 1. Participant Flow Chart

Supplemental Table 1. Food Patterns Equivalents Database Components

Supplemental Figure 1. Participant Flow Chart

Excluding participants with any missing values on age, sex, race/ethnicity, education level, ratio of family income to poverty, smoking status, physical activity, medication use, blood pressure, body mass index, blood glucose, glycosylated hemoglobin, lipid profiles, and C-reactive protein (*n*=7,068)

Training data

*n*=3,401

Test data

*n*=377

Participants with complete information on covariates

*n*=3,778

Participants with diet and pyridoxal 5'-phosphate data

*n*=5,528

Participants with diet and pyridoxal 5'-phosphate data

*n*=5,318

Participants aged ≥ 20 years

*n*=6,218

Participants aged ≥ 20 years

*n*=5,935

NHANES 2007-2008

*n*=9,762

NHANES 2009-2010

*n*=10,253

### Supplemental Table 1. Food Patterns Equivalents Database Components

| FPED component | Foods and Units |
| --- | --- |
| Total Fruit | Total intact fruits (whole or cut) and fruit juices (cup eq.) |
| Citrus, Melons, and Berries | Intact fruits (whole or cut) of citrus, melons, and berries (cup eq.) |
| Other Fruits | Intact fruits (whole or cut); excluding citrus, melons, and berries (cup eq.) |
| Fruit Juice | Fruit juices, citrus and non-citrus (cup eq.) |
| Total Vegetables | Total dark green, red and orange, starchy, and other vegetables; excludes legumes (cup eq.) |
| Dark Green Vegetables | Dark green vegetables (cup eq.) |
| Total Red and Orange Vegetables | Total red and orange vegetables (tomatoes and tomato products + other red and orange vegetables) (cup eq.) |
| Tomatoes | Tomatoes and tomato products (cup eq.) |
| Other Red and Orange Vegetables | Other red and orange vegetables, excluding tomatoes and tomato products (cup eq.) |
| Total Starchy Vegetables | Total starchy vegetables (white potatoes + other starchy vegetables) (cup eq.) |
| Potatoes | White potatoes (cup eq.) |
| Other Starchy Vegetables | Other starchy vegetables, excluding white potatoes (cup eq.) |
| Other Vegetables | Other vegetables not in the vegetable components listed above (cup eq.) |
| Beans and Peas | Beans and peas (legumes) computed as vegetables (cup eq.) |
| Total Grains | Total whole and refined grains (oz. eq.) |
| Whole Grains | Grains defined as whole grains and contain the entire grain  kernel ― the bran, germ, and endosperm (oz. eq.) |
| Refined Grains | Refined grains that do not contain all of the components of the entire grain kernel (oz. eq.) |
| Total Protein Foods | Total meat, poultry, organ meat, cured meat, seafood, eggs, soy, and nuts and seeds; excludes legumes (oz. eq.) |
| Total Meat, Poultry, and Seafood | Total of meat, poultry, seafood, organ meat, and cured meat (oz. eq.) |
| Meat | Beef, veal, pork, lamb, and game meat; excludes organ meat and cured meat (oz. eq.) |
| Cured Meat | Frankfurters, sausages, corned beef, and luncheon meat that are made from beef, pork, or poultry (oz. eq.) |
| Organ Meat | Organ meat from beef, veal, pork, lamb, game, and poultry (oz. eq.) |
| Poultry | Chicken, turkey, Cornish hens, duck, goose, quail, and pheasant (game birds); excludes organ meat and cured meat (oz. eq.) |

| FPED component | Foods and Units |
| --- | --- |
| Seafood High in *n*-3 Fatty Acids | Seafood (finfish, shellfish, and other seafood) high in *n*-3 fatty acids (oz. eq.) |
| Seafood Low in *n*-3 Fatty Acids | Seafood (finfish, shellfish, and other seafood) low in *n*-3 fatty acids (oz. eq.) |
| Eggs | Eggs (chicken, duck, goose, quail) and egg substitutes (oz. eq.) |
| Soy Products | Soy products, excluding calcium fortified soy milk and mature soybeans (oz. eq.) |
| Nuts and Seeds | Peanuts, tree nuts, and seeds; excludes coconut (oz. eq.) |
| Beans and Peas | Beans and Peas (legumes) computed as protein foods (oz. eq.) |
| Total Dairy | Total milk, yogurt, cheese, and whey. For some foods, the total dairy values could be higher than the sum of milk, yogurt and cheese because Miscellaneous dairy component composed of whey which is not included in FPED as a separate variable. (cup eq.) |
| Milk | Fluid milk, buttermilk, evaporated milk, dry milk, and calcium fortified soy milk (cup eq.) |
| Yogurt | Yogurt (cup eq.) |
| Cheese | Cheeses (cup eq.) |
| Oils | Fats naturally present in nuts, seeds, and seafood; unhydrogentated vegetable oils, except palm oil, palm kernel oil, and coconut oils; fat present in avocado and olives above the allowable amount; 50% of fat present in stick and tub margarines and margarine spreads (grams) |
| Solid Fats | Fats naturally present in meat, poultry, eggs, and dairy (lard, tallow, and butter); hydrogenated or partially hydrogenated oils; shortening, palm, palm kernel and coconut oils; fats naturally present in coconut meat and cocoa butter; and 50% of fat present in stick and tub margarines and margarine spreads (grams) |
| Added Sugars | Foods defined as added sugars (tsp. eq.) |
| Alcoholic Drinks | Alcoholic beverages and alcohol (ethanol) added to foods after cooking (no. of drinks) |
